# Quantification of groundwater discharge into a shallow coastal lagoon applying a multi-tracer approach

**DOI:** 10.1007/s10661-023-11244-3

**Published:** 2023-04-21

**Authors:** Michael Schubert, Kay Knoeller, Jan Scholten, L. Walter Daesslé, Mauricio M. Reyes Bravo, Efraín M. Chávez Solís

**Affiliations:** 1 Helmholtz Centre for Environmental Research, UFZ Leipzig/Halle, Halle, Germany; 2 Coastal Geology and Sedimentology, Kiel University, Kiel, Germany; 3 Instituto de Investigaciones Oceanológicas, Universidad Autónoma de Baja California, Ensenada, Mexico

**Keywords:** Submarine groundwater discharge, Coastal lagoons, Tracer, ^222^Rn, ^223^Ra, ^224^Ra, δ^18^O, δ^2^H

## Abstract

In many cases, shallow coastal lagoons are, on the one hand, vulnerable habitats for birds and marine ecosystems and, on the other hand, threatened by discharging nutrient-laden surface waters and groundwater. In particular, the localization and quantification of submarine groundwater discharge (SGD) is of key concern in this regard. The presented study aimed at investigating SGD into a vulnerable coastal lagoon that is strongly impacted by evaporation applying a multi-tracer approach. The joint application of radionuclides (^222^Rn, ^223^Ra, ^224^Ra), stable water isotopes (δ^18^O, δ^2^H) and the water salinity as environmental water tracers allowed evaluating the suitability of the individual parameters in this specific type of environment. Whilst stable isotope and salinity data were difficult to construe in terms of SGD occurrence due to the intense impact of evaporation, a radon mass balance allowed localising SGD areas within the lagoon and quantifying the related SGD flux rates. In addition, a ^224^Ra/^223^Ra ratio analysis revealed information on the apparent age of the discharged groundwater, and hence on the flushing intensity of the lagoon. Besides these site-specific results, the study allowed the following general conclusions regarding the suitability of the applied tracers: (i) we verified the suitability of a radon mass balance approach for proving/disproving SGD occurrence and quantifying SGD fluxes in shallow coastal lagoons strongly impacted by evaporation; (ii) we showed that the impact of evaporation may impede the use of water stable isotope and salinity data as SGD indicators in such specific environments; (iii) we demonstrated that the tidal impact on a lagoon water body during a sampling campaign can be compensated by adapting sampling schedule and cruise track to the tidal cycle.

## Introduction

As a result of the increasing urbanisation of coastlines worldwide, sustainable coastal water management is of rising concern. Related research activities focus on the vulnerability of (i) terrestrial coastal aquifers, (ii) estuaries and lagoons and (iii) the shallow coastal sea immediately adjacent to the shore. Since the terrestrial aquifers and the named coastal waters are strongly interconnected, investigation of groundwater/seawater interaction is of major relevance in this context. In particular, investigating the flux of groundwater (and associated dissolved matter) from the terrestrial aquifer to the coastal waters, i.e. submarine groundwater discharge (“SGD”), is a focal point of research (e.g. Burnett et al., 2006; Taniguchi et al., 2001, 2019). Two potential environmental threats justify its relevance, namely (i) the detrimental impact of nutrient- and/or contaminant-laden SGD on the coastal water quality, an aspect that is of particular significance along coastlines with intense urbanisation and agriculture, and (ii) the loss of freshwater to the coastal sea, an issue that is mainly of concern in climate zones with (seasonally) low groundwater recharge and associated limited availability of freshwater (e.g. Rocha et al., 2016; Slomp & Van Cappellen, 2004).

The study presented in this paper was executed in a coastal area that is characterised by both substantial human impact on the groundwater quality and water shortage (Bahia de Todos Santos, Baja California, Mexico). The site-specific aim of the study was (i) to prove/disprove SGD both to the coastal sea and to a vulnerable coastal lagoon, (ii) to quantify SGD flux both to the coastal sea and to the lagoon and (iii) to estimate the residence time of the discharged groundwater within the lagoon. For answering the related questions, a field campaign was executed in the course of which water samples from the lagoon, the coastal sea and the groundwater were taken and analysed for natural radionuclides (^222^Rn, ^223^Ra, ^224^Ra and ^226^Ra) and stable water isotopes (δ^18^O, δ^2^H) as well as the parameter salinity. All these tracers and indicators are characterised by a strong gradient at the groundwater/seawater interface, thus allowing the investigation of groundwater/seawater interaction, in particular SGD (e.g. Burnett et al., 2008; Petermann et al., 2018; Schubert et al., 2015). Besides the site-specific results, the paper presents general conclusions regarding the individual applicability of the chosen tracers and their combined use for the specific setting of a shallow coastal lagoon that is strongly impacted by evaporation.

## Materials and methods

### Study site

**Fig. 1:**
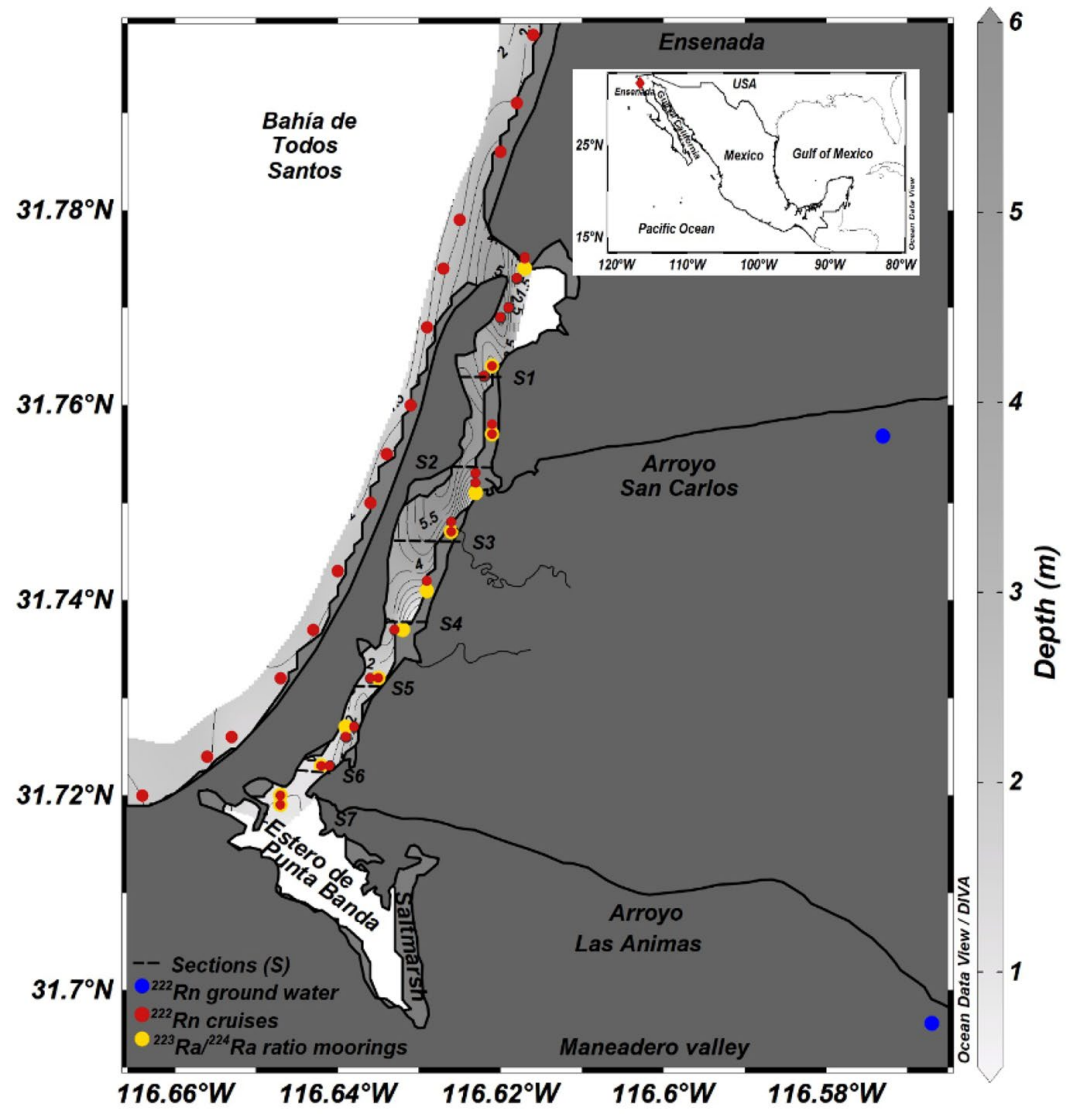
Map of Estero de Punta Banda, Baja California, Mexico, showing the sampling locations for radon (raw data; see section “Processing of the radon mapping data”) and ^223^Ra/^224^Ra, the two sampled groundwater wells and the seven 1-km subsections (“S1”– “S7”) of the lagoon. Throughout the text, the sampling locations within Bahia de Todos Santos (i.e. west of the sandbar) are referred to as “coastal sea”, and the locations within Estero de Punta Banda as “lagoon”. The scale does only allow to illustrate a maximum depth of 6 m within the Estero de Punta Banda; however, a steep slope maximum depth of 10 m is present in the north as discussed in the text

The study area is located on the west coast of the Baja California peninsula, in the southern part of Bahia de Todos Santos, ca. 10 km south of the city of Ensenada (Fig. 1). The actual study site was an about 10-km-long stretch of shoreline including a connected shallow tide-influenced coastal lagoon (Estero de Punta Banda), which in 2006 was declared a Ramsar site for its ecological importance and relevance of its conservation (CONANP, 2012). The coastal aquifer of concern is the Maneadero aquifer, which has an extension of ca. 130 km^2^ and sits underneath the Maneadero valley contained within an alluvial fan composed mainly of Quaternary fluvial and marine deposits, delimited to the south by the Agua Blanca fault, and to the west by the Estero de Punta Banda. The San Carlos and Las Ánimas creeks are the main perennial watersheds that drain into the basin along 1879 km^2^. The annual runoff of the San Carlos creek averages 10 Mm^3^ and of the Las Ánimas 23 Mm^3^ (Gilabert-Alarcón et al., 2018). Due to the resulting high water demand, the Maneadero aquifer is subject to groundwater over-abstraction of 5 Mm^3^ per year (CONAGUA, 2020) and probably as high as 17 Mm^3^ (DOF, 2015). The hydraulic gradient is significantly influenced by intense water pumping for domestic supply adjacent to both riverbeds, inducing a flow gradient from the shore into the aquifer during prolonged dry periods. Although seawater influence is not detected in these abstraction sites, the extension of the seawater intrusion prism reaches ca. 3 km into the aquifer (*cf*. Gilabert-Alarcón et al., 2018). No information exists regarding the detailed influence the tidal prism has on the aquifer.

The Maneadero valley is characterised by intense agricultural activity (growing vegetables, flowers and fodder crops), cattle raising and the production of dairy products. The cultivable area covers about 40 km^2^ with irrigation relying mainly on groundwater exploitation. In addition, the Maneadero aquifer is one of the most important water sources to the city of Ensenada and the town of Maneadero. The groundwater is produced from 350 wells, which are mostly 25–60 m deep (Daesslé et al., 2014). The well sampled for this study near San Carlos creek is ca. 100 m deep, whilst the one sampled from Las Ánimas is ca. 60 m deep.

At the same time, the groundwater of the Maneadero aquifer is suffering substantial pollution by nitrate. The pollution (N-NO_3_ > 40 mg/L) is caused by overfertilization, by the lack of domestic drainpipes and by managed aquifer recharge (MAR) activities, i.e. the re-infiltration of secondary treated wastewater originating from the city of Ensenada (Gilabert-Alarcón et al., 2018). MAR is in operation since 2014 for supporting the aquifer’s groundwater balance. The treated wastewater is discharged with a rate of about 400 L/s directly into the beds of the two ephemeral rivers San Carlos and Las Animas (*cf*. Fig. 1), as well as used to irrigate flower crops.

A key feature of the study area is the coastal lagoon Estero de Punta Banda, which is located behind a sand bar mainly composed of beach sand and dunes, that stretches for about 8 km parallel to the coastline (Fig. 1). The two abovementioned ephemeral watersheds drain into the lagoon. Arroyo San Carlos drains an area of 815 km^2^ with a length of 60 km. Arroyo Las Animas drains an area of 980 km^2^ with a length of 58 km. However, Estero de Punta Banda can be named an estuary only during the most extreme and seldom rainy winters as it receives drainage from the two creeks only during this season (November–March). During the main time of the year, the waterbody is more appropriately characterised as lagoon. The central canal of the lagoon reaches water depths of up to 10 m but is very shallow at the lagoon’s head (about 1 m; Ortiz et al., 2003; Poumian-Tapia et al., 1997). Impacted by the tides (with a mean tidal range of 1.04 m), the lagoon shows a mean water volume of about 3 × 10^6^ m^3^. The central canal and its (periodically flooded) surrounding tidal flats cover an overall area of about 12 km^2^.

The annual average precipitation of about 230 mm/a is considerably exceeded by evaporation from the lagoon’s surface (about 1000 mm/a; Smith et al., 1997). Hence, the lagoon’s water budget is primarily balanced by seawater inflow. This precipitation/evaporation disproportion results in a high water salinity increasing from the mouth to the head of the lagoon (Celis-Ceseña & Alvarez-Borrego, 1975). No formal studies on SGD to Estero de Punta Banda exist so far. Smith et al. (1997) assumed no groundwater inflows (nor stream runoff) at all for the dry season. Submarine discharge of treated wastewater, which is originating from the abovementioned MAR activity close to the city of Ensenada, into the lagoon might take place, though.

### On-site activities

The aim of the study was the localization and quantification of groundwater discharge into the coastal sea and the shallow lagoon based on a multi-tracer approach applying the tracers radon (^222^Rn), short-lived radium (^223^Ra/^224^Ra) and water stable isotopes (δ^2^H/δ^18^O) as well as the parameter water salinity. The related on-site sampling activities included (i) radon mapping campaigns both in the lagoon and the coastal sea aiming at localising and quantifying groundwater discharge based on radon mass balance calculations, (ii) radon detection in the hydraulically connected groundwater for mass balance endmember definition, (iii) ^226^Ra detection in the lagoon sediments for quantification of diffusive radon input into the lagoon, (iv) ^223^Ra/^224^Ra detection in the lagoon water for water age distribution mapping within the lagoon and (v) δ^2^H/δ^18^O and water salinity mapping both in lagoon and coastal sea aiming at supporting the radon mass balance results. All activities took place during the dry season, namely in May 2022 (^222^Rn, salinity and δ^18^O/δ^2^H in the lagoon incl. groundwater endmember) and in August 2022 (radium sampling in the lagoon; ^222^Rn and δ^18^O/δ^2^H in the coastal sea).

### Radon mapping in lagoon and coastal sea

Mapping of the radon distribution both in the lagoon and the 10-km stretch of coastal sea west of it was done directly on site aboard a slowly cruising vessel by radon extraction from a continuous water pump stream applying a membrane extractor (MiniModule^®^, Membrana GmbH) (Schmidt et al., 2008). For the purpose, coastal sea/lagoon water was continuously pumped from about 0.5 m water depth with a pumping rate of about 2 L/min. The extracted radon was measured at once of by means of mobile radon-in-air detection equipment (RAD-7, Durridge, Billerica, MA, USA) set to a 10-min counting cycle. The approach had been proven suitable for the purpose during numerous field campaigns (e.g. Petermann et al., 2018). For radon mapping within the lagoon, two detection setups (i.e. two water pumps, two membrane extractors and two RAD7 radon monitors) were run in parallel. Due to a malfunctioning water pump, only one detection setup could be used on the coastal sea. All detected radon-in-air concentrations were converted into the associated radon-in-water concentrations by allowing for the temperature- and salinity-dependent water/air radon partitioning coefficient (Schubert et al., 2012). Both temperature and salinity were mapped simultaneously to radon.

The radon measurement cruises took place along a 7-km transect within the lagoon (as illustrated in Fig. 1) and along a 10-km stretch within the coastal sea west of the lagoon’s sand bar, the latter starting at the Punta Banda Peninsula and ending about 2 km north of the lagoon’s mouth (distance to the shoreline < 100 m). The vessel was cruising with a speed of about 4 km/h keeping the water depth at around 1 m. GPS was used for recording all cruise tracks and positions of radon records. Water salinity and water depth were constantly monitored using a CTD probe. The water temperature was recorded at the outlet of the radon MiniModule^®^ membrane extractor.

### Estimating the radon endmember in groundwater

Calculating a radon mass balance of the lagoon water body including all radon sources and sinks requires a radon-in-groundwater endmember as SGD source term. For determination of the radon-in-groundwater endmember, groundwater samples were taken from two wells located adjacent to both riverbeds ca. 4 km inland (Fig. 1). All samples were taken after purging of the wells. Radon concentrations were measured with a mobile radon-in-air monitor (AlphaGuard PQ 2000; Saphymo, Frankfurt, Germany) by degassing the radon from 250 mL water samples in a closed air loop as described in Schubert et al. (2006). The actual water sampling was done in a way that guarantees minimum water/air contact and minimum water turbulence in order to avoid radon loss during sampling. All detected radon-in-air concentrations were converted into radon-in-water concentrations by allowing for the water/air radon partitioning coefficient.

### Estimating diffusive radon input based on the ^226^Ra activity concentration in lagoon sediments

Diffusive radon flux from the uppermost sediment layer into the lagoon waterbody was assessed based on the equilibrium radon concentration of the lagoon’s sediment porewater. For determination of the latter, sediment material was collected from the sediment surface and its radium activity concentration measured by γ-spectroscopy. Measurement was done by an ORTEC-Gamma-X HPGe coaxial low-energy n-type detector with an active volume of 39 cm^3^ and a 0.5-mm beryllium window. Sediment samples were taken at three representative locations, dried, weighted and measured twice each for 24 h. Detector and measuring geometry (applying cylindrical radon-tight capsules of 32 cm^3^) were calibrated using the certified IAEA reference material RGU-1. The radium activity concentration was determined based on the distinct gamma emission energies of the short-lived radon progeny ^214^Pb (295.2 and 351.9 keV) and ^214^Bi (609.3 and 1120.3 keV) with a mean standard deviation of 5%. For ensuring decay equilibrium between radium and the short-lived radon progeny, the samples were stored before measurement for 27 days (seven ^222^Rn half-lives) in the radon-tight capsules.

### ^223^Ra / ^224^Ra detection in lagoon water

The half-lives of the naturally occurring radium nuclides ^223^Ra and ^224^Ra (11.1 days and 3.6 days, respectively) result in a decrease of the (unsupported) ^224/223^Ra activity ratio with a combined half-life of 5.35 days. Hence, the ^224/223^Ra activity ratio detected in water samples can be used as water age indicator (“radium age”) (Moore, 2000; Garcia-Orellana et al., 2021). Still, dissolved radium tends to get strongly adsorbed onto the mineral aquifer matrix if the concentration of competing cations in the groundwater is low. Hence, radium concentrations are generally negligible in fresh groundwater. In contrast, saline and brackish porewaters show elevated radium concentrations. As a result, the ^224/223^Ra activity concentration ratio detected in the coastal sea can be used as measure of the mean residence time of porewater that was freshly discharged from the marine sediments into the coastal seawater (i.e. in the given case the lagoon) (Burnett et al., 2008; Moore, 2000).

For determination of the ^223^Ra/^224^Ra ratio, moorings were placed at twelve points equidistantly distributed in the lagoon (*cf*. Fig. 1). The dissolved radium was allowed to naturally adsorb (i.e. pre-concentrate) onto MnO_2_-coated fibres that were placed in mesh bags and attached to the moorings 1 m below the water surface for 24 h (Moore, 1976). After collection of the radium-laden fibre bags, ^224^Ra and ^223^Ra activities were determined by means of a delayed coincidence counting system (RaDeCC; Moore & Arnold, 1996). For efficiency calibration of the RaDeCC, a ^232^Th standard was used (for ^224^Ra). The efficiency calibration for ^223^Ra was performed following Moore and Cai (2013). Counting errors were propagated following Garcia-Solsona et al. (2008).

### δ^2^H/δ^18^O detection in water samples

Selected water samples were taken from the lagoon, the coastal sea and groundwater and analysed for their water stable isotope signatures (δ^2^H and δ^18^O). The groundwater samples were taken from the same three wells that were sampled for the radon groundwater endmember. Seawater samples were taken at the twelve mooring locations within the lagoon and at 24 equidistant locations along the coastal radon profile.

The stable isotopic composition of the samples was determined in the laboratory by the H_2_O-H_2_ equilibration method (^2^H) with an analytical precision of ± 1.0‰ and the H_2_O-CO_2_ equilibration method (^18^O) with an analytical precision of ± 0.1‰. Water stable isotope signatures of the treated wastewater were available from an earlier study (Gilabert-Alarcón et al., 2018). All δ^2^H/δ^18^O datasets are expressed as delta notations relative to the Vienna Standard Mean Ocean Water (VSMOW).

### Processing of the radon mapping data

For SGD quantification into the lagoon, a radon mass balance was calculated for the lagoon’s waterbody. The mass balance generally assumes a steady-state radon inventory within the lagoon [Bq]. For data processing, the 7-km profile along the lagoon’s main canal was sub-divided into seven subsections, each 1 km in length (“S1”– “S7”). The radon inventory was determined individually for each of the seven subsections. Each of the seven radon inventories is balanced by radon sinks and sources. The sinks include atmospheric degassing [Bq m^−2^ d^−1^] and radon decay [Bq m^−3^ d^−1^]; the sources are diffusive radon input from the sediment [Bq m^−2^ d^−1^] and radon input associated to SGD (i.e. the sought-after parameter, [Bq m^−3^ d^−1^]). Radioactive radon production within the lagoon’s water column and discharge of the two ephemeral rivers into the lagoon could be neglected as source terms due to the negligible ^226^Ra activity (i.e. the parent isotope of ^222^Rn) in the seawater and the absence of any river discharge during the season. After quantifying all measurable sources (i.e. diffusive input) and sinks (i.e. degassing and decay), the positive radon flux that is required to sustain a balanced radon inventory was calculated for each of the seven subsections (and thus for the total lagoon) and attributed to SGD. Based on the groundwater endmember concentration, this SGD-related radon flux was finally converted into the actual SGD water flux.

The following paragraphs briefly introduce the corrections that have to be applied to radon data that were recorded whilst cruising on lagoon and coastal sea. However, since the radon concentrations recorded in the coastal sea, i.e. west of the lagoon’s sand bar, were too low to indicate any SGD there, no in-detail assessment of the recorded coastal sea radon data was executed. Hence, the listed corrections were applied to the lagoon data series only.

(i) Location correction: Each radon detection point was shifted 5 min backwards in order to position each data point to the middle of the cruise track section that was covered during the corresponding 10-min counting cycle.
(ii) Response delay correction: Since the RAD7 radon detector is not counting ^222^Rn decays directly but the decays of the radon progeny ^218^Po, the radon time series required a response delay correction. Besides the delayed decay equilibrium between ^222^Rn and ^218^Po, the applied correction allowed for the water → air transfer kinetics of radon during the enforced degassing process. Experiences with the applied detection setup and procedure suggested a response delay correction of 10 min (Petermann & Schubert, 2015).
(iii) Correction of tidal influence: The radon activity concentrations recorded in the lagoon had to be corrected for the influence of the varying tide, i.e. for the influence of the periodically occurring tidal current. That influence was allowed for by cruising the lagoon profile twice, once from mouth to head starting at about low tide and once backwards from head to mouth ending at about high tide. The radon values resulting from the two cruises were averaged for each of the seven subsections, thereby levelling out the tidal influence.
(iv) Radon degassing correction: Radon degassing from the lagoon’s water surface was quantified based on an empirical model developed by MacIntyre et al. (1995), which defines the actual wind speed as the main influential parameter for the degassing loss. The approach presumes that no intense storm events had occurred in the days prior to the actual sampling campaign because the radon inventory would have needed time to regenerate after such an event (Petermann et al., 2018). That could be confirmed for the sampling campaign discussed here. Comparable to the correction of the tidal influence, the change in wind speed during the survey on the lagoon (very low wind at the beginning and wind speeds around 7 m/s at the end of the cruise) was accounted for by cruising the profile twice, mouth-head during low winds in the morning and head-south during increasing winds in the afternoon.
(v) Radon decay correction: Radon decay in the water is solely a function of the ^222^Rn decay constant and the activity concentration. It was allowed for based on the decay equation.

Besides the mentioned corrections of the individual values, diffusive radon flux from the lagoon sediment had to be accounted for as radon source that is not associated to SGD. Diffusive radon flux is mainly driven by the radon concentration gradient between the surface water body (i.e. the lagoon) and the porewater of the upper sediment layer. Whilst the first was measured directly as described above, the latter was calculated based on the ^226^Ra activity concentrations of three representative samples that were collected from the sediment top layer. Based on the concentration gradient (and the radon diffusion coefficient in the sediment), the diffusive radon flux was calculated as shown by Corbett et al. (1998).

## Results and discussion

### Radon in coastal sea, lagoon, porewater and groundwater

Along the 10-km coastal profile within the coastal sea west of the lagoon’s sand bar (*cf*. Fig. 1), the radon activity concentrations were found to be close to the detection limit of the applied monitoring setup. Due to the low concentrations (and due to the abovementioned malfunctioning water pump, which allowed using only one detection setup along the coastal profile), the uncertainty of the recorded data is high. Values that correspond to the offshore background (around 7 ± 11 Bq/m^3^) were localised in the middle of the profile. Slightly elevated concentrations (15 ± 13 Bq/m^3^) detected at the southern end of the profile, i.e. off the head of the lagoon, indicate minor SGD there. The indicated SGD is potentially associated to the hydrogeological setting of the ephemeral river Arroyo Las Ánimas. (*cf*. Fig. 1). A paleo-channel of the river might provide a preferential flow path for groundwater and promote groundwater flow there even though the river was dry during the sampling campaign. Slightly elevated concentrations were also detected close to the lagoon’s mouth (14 ± 11 Bq/m^3^). They do also indicate minor SGD (potentially associated to the ephemeral river Arroyo San Carlos) as well as discharge of brackish surface water from the lagoon into the Bahia de Todos Santos. However, the low detected concentrations and the high detection uncertainty of the mapping results along the 10-km coastal profile do not allow any reliable quantitative evaluation of SGD into Bahia de Todos Santos; the SGD flux rates are too small for sound quantification.

**Fig. 2:**
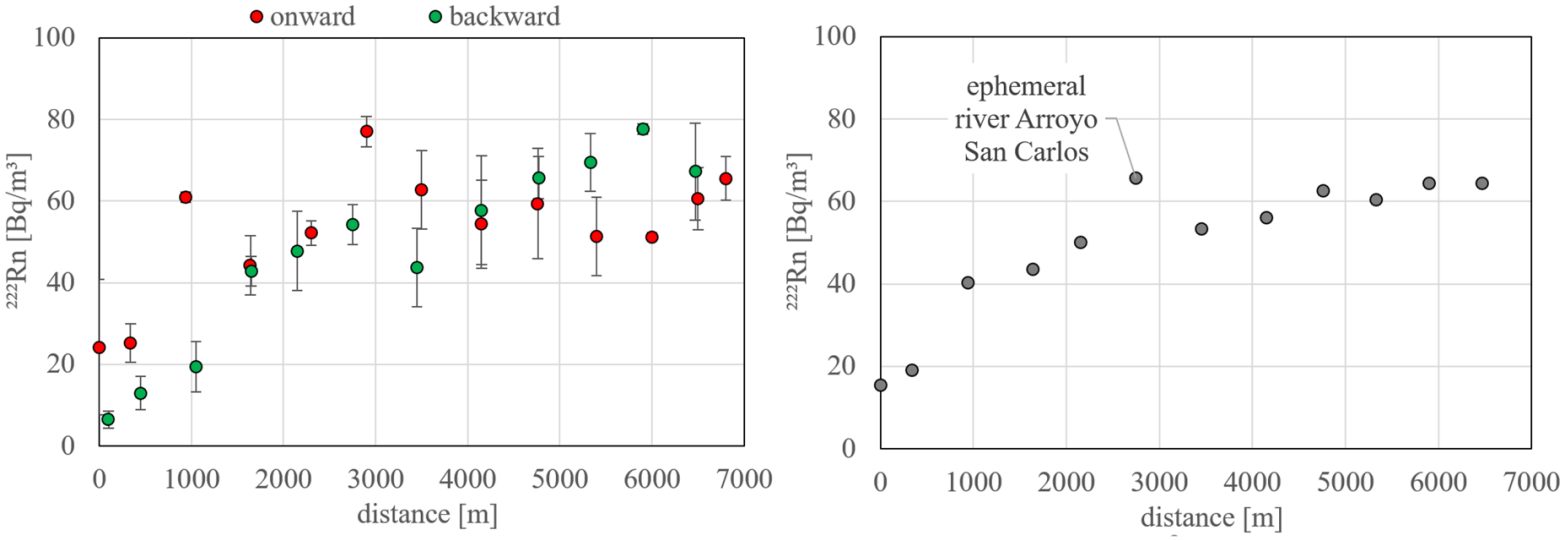
Radon distribution pattern within the lagoon’s main canal starting at the mouth (0 m) ending at the lagoon’s head (7000 m) as illustrated in Fig. 1. **Left** Onward and backward cruises plotted individually. **Right** Mean values of (in each case) two individual readings recorded during the onward and the backward cruise, respectively, at adjoining locations

The situation is different within the lagoon. Figure 2a displays the radon concentrations recorded during both onward and backward cruise individually. The detection uncertainty of the mapping results is lower than in the coastal sea because two detection setups were run in parallel in the lagoon. As outlined above, the sampling vessel set off around low tide at the mouth of the lagoon, cruised to its head with the tide rising and returned to the mouth approaching high tide. Due to the tidal current that is entering the lagoon from the open bay with the rising tide (low in radon), the radon activity in the vicinity of the mouth was slightly decreased during the end of the cruise compared to its onset. This decrease is also reflected in the simultaneously recorded salinity values (*cf*. Fig. 5). Another reason for differences between onward and backward cruise data points located at almost similar distances from the lagoon’s mouth is the slightly varying onward/backward tracks of the vessel in the lagoon’s main canal. An immediate homogenous mixing of lagoon water and tidal current in the canal cannot be expected.

Figure 2 right displays the same values as Fig. 2 left but averages the readings of data points recorded during the onward and the backward cruise at adjoining locations (*n* = 2). The plot reveals a significant concentration gradient between the lagoon’s mouth (around 15 Bq/m^3^; i.e. comparable to the value detected in the coastal sea close to the mouth) and its head (around 65 Bq/m^3^). Generally, the elevated concentrations suggest that the lagoon receives groundwater discharge (elevated in radon). The mapped radon concentrations peak locally at a position that seems to be associated to the bed of the ephemeral river Arroyo San Carlos, i.e. to a hydrogeological setting that might be favourable for groundwater flow.

For setting up a radon mass balance, radon concentrations had to be converted into radon inventories. Using the lagoon’s bathymetry, the mean water volumes of the seven subsections S1 to S7 were calculated. The resulting volumes [m^3^] and the associated mean radon activities [Bq/m^3^] of each subsection allowed calculating average radon inventories [Bq] for each subsection. Based on the groundwater radon endmember revealed by the groundwater samples (23 kBq/m^3^), the subsection-specific SGD rates [m^3^/d], which are required to support the subsection-specific radon inventories, were concluded. Both diffusive radon input from the sediment and radon loss by degassing from the water surface were considered in this mass balance calculation as follows.

Diffusive radon input: The diffusive radon input was calculated for each subsection based on the sediment parameters radium activity concentration as detected by γ-spectroscopy (*A*_Ra_ = 18.1 Bq/kg), emanation coefficient (*ε* = 0.3; based on a set of typical values published by Nazaroff & Nero, 1988), dry density as measured by weighing (*δ* = 1400 kg/m^3^) and porosity (using *n* = 0.6 based on the water content of the samples) (Eq. 1).

1 $${C}_{\mathrm{Eq}}=\frac{{A}_{\mathrm{Ra}}\times \varepsilon \times \delta }{n}$$

The calculation resulted in an equilibrium porewater radon concentration of the upper sediment layer of *C*_Eq_ = 12.7 kBq/m^3^.

Based on literature data (Corbett et al., 1998; Nazaroff & Nero, 1988), a radon diffusion coefficient in the sediment of *D*_*s*_ = 5 × 10^−10^ m^2^/s was assumed. Resulting from it (and the ^222^Rn decay constant *λ* = 2.1 × 10^−6^ s^−1^), a diffusive radon flux from the sediment of *J*_Rn_ = 35 Bq/m^2^d was calculated (Eq. 2).

2 $${J}_{\mathrm{Rn}}=\sqrt{\lambda {D}_{s}}{\times (C}_{\mathrm{Eq}}-{C}_{0})$$

Radon loss by degassing: For calculating the radon loss by degassing from the water surface, an average wind speed of 3 m/s was assumed. This average wind speed represents the situation encountered during the survey (with winds steadily increasing from about 1 m/s at the beginning to about 7 m/s at the end of the cruise). Radon in the air was measured whilst purging the radon detectors before and after each radon mapping survey. The radon in air concentrations was negligible. The resulting radon flux from the water surface was calculated as outlined by MacIntyre et al. (1995) and amounts to 26 Bq/m^2^d.

**Fig. 3:**
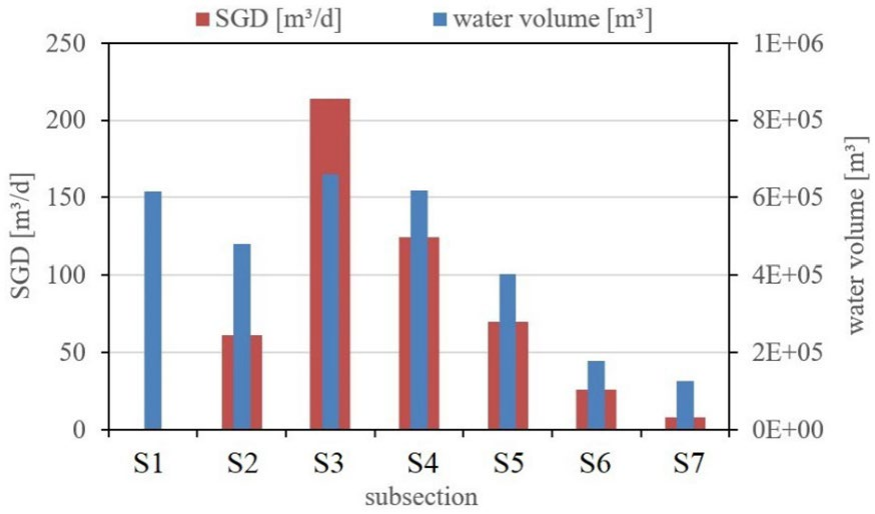
SGD and water volume of the seven subsection of the lagoon, each 1 km long

Summing up all known sink and source terms for each of the seven subsections of the lagoon (i.e. radon loss by decay, radon loss by degassing and radon input by diffusion) reveals the subsection-specific SGD-borne radon input [Bq/d] that is required to balance the individual steady-state radon inventory. The associated SGD flux rates [m^3^/d], calculated based on the radon groundwater endmember (23 kBq/m^3^), are displayed in Fig. 3. As shown, highest SGD flux rates were deduced in subsections S3 and S4, and lowest in subsections S1 and S7. The SGD-borne radon input required for balancing the radon inventory for the complete lagoon amounts to 11.1 kBq/d being equivalent to an overall SGD rate of 485 m^3^/d. This daily input corresponds to about 0.02% of the total water volume of the lagoon.

### ^223^Ra and ^224^Ra in lagoon water

For mapping the radium age distribution pattern within the lagoon (and for assessing the intensity of the lagoon’s tidal flushing), the highest detected ^224/223^Ra ratio was defined as initial value, i.e. as representative for sediment porewater freshly discharged into the lagoon’s water body. The highest detected ^224/223^Ra ratio amounted to 21.2. It was detected at buoy located at km 5.8 in subsection S6. Based on this initial value and the combined ^224/223^Ra half-life (5.35 days), all obtained ^224/223^Ra ratios were transferred into radium ages at the sampling location (11 buoys; *cf*. Fig. 1). The radium ages represent the mean apparent water ages of the discharged porewater at the eleven sampling locations recorded over a 24-h exposure time.

**Fig. 4:**
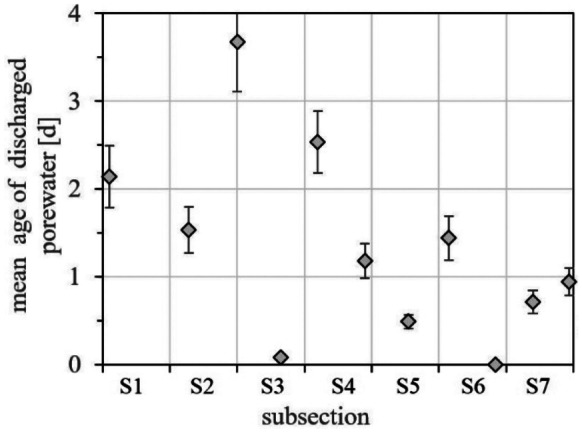
SGD residence times in the lagoon based on ^224/223^Ra ratios

Figure 4 displays the resulting mean apparent water ages at the eleven sampling locations. The figure reveals that the lowest mean water ages of up to 1.5 days were detected in subsections S5, S6 and S7, i.e. at the head of the lagoon and in its central section. Even though the determined SGD flux rate is rather low in this part of the lagoon (*cf*. Fig. 3), this observation is reasonable because the net migration of discharged groundwater (incl. the radium containing porewater) is towards the mouth of the lagoon, thus accumulating higher water ages close to the mouth. Freshly discharged porewater was also detected in subsection S3 (km 2.7). The location corresponds to the local radon peak within the lagoon indicating relatively intense SGD (*cf*. Fig. 2b). Generally, the radon data indicated the most intense SGD flux in subsection S3 (*cf*. Fig. 3), a result that is in accordance with the low mean water age localised there.

Relatively high apparent ages of the discharged porewater (ages between 1.4 and 3.7 days) were found in subsections S1 to S3. The “oldest” water (3.7 days) was detected in 2 km distance from the lagoon’s mouth (subsection S2). This is the section of the inner lagoon that shows the greatest water depth (up to 10 m), thus hampering tidal water exchange.

Generally, the revealed maximum mean water ages of around 4 days indicate that the tidal flushing of the lagoon is intense. The result is in accordance with earlier assumptions of a noticeable effect of the tides on Estero de Punta Banda, with up to 60% of the water possibly evacuated in one tidal cycle (Alvarez, 1992).

### δ^2^H, δ^18^O and salinity in water samples

**Fig. 5:**
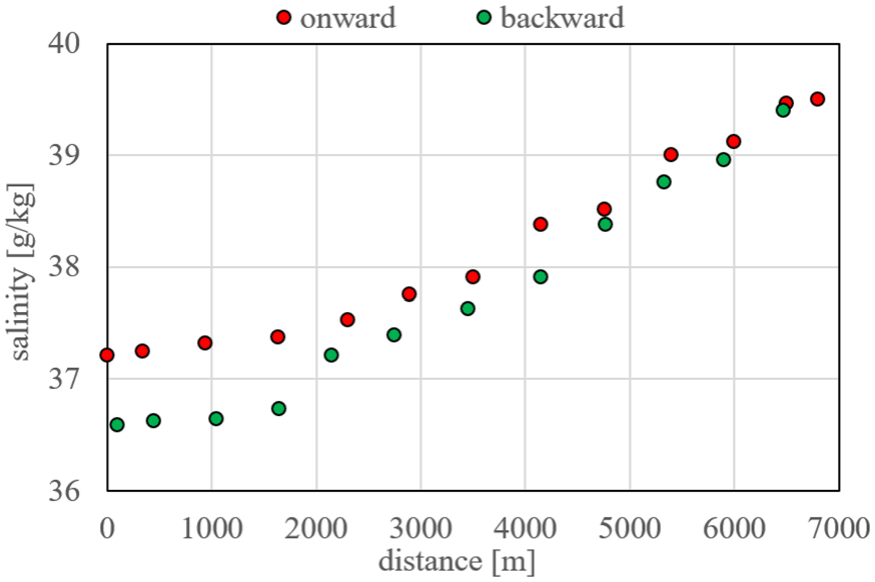
Water salinity mapped within the lagoon revealing a strong evaporation impact and the range of tidal influence

Comparison of the water stable isotope distribution patterns detected in the coastal sea and the lagoon shows a strong evaporation impact on the lagoon’s waterbody. This evaporation impact is also revealed by the water salinity distribution mapped within the lagoon, which rises significantly with distance from its mouth (Fig. 5). The water salinity in Bahia de Todos Santos is around 31.3 g/kg. In contrast, the salinity in the lagoon was detected to be around 37.0 g/kg at its mouth and 39.5 g/kg at its head. Close to the mouth, a slightly lower salinity was detected during the return cruise because of the rising tide, i.e. the incoming, less saline seawater. This impact of the tidal current is most pronounced within subsections S1 and S2 and also reflected in the radon data (*cf*. Fig. 2a).

**Fig. 6:**
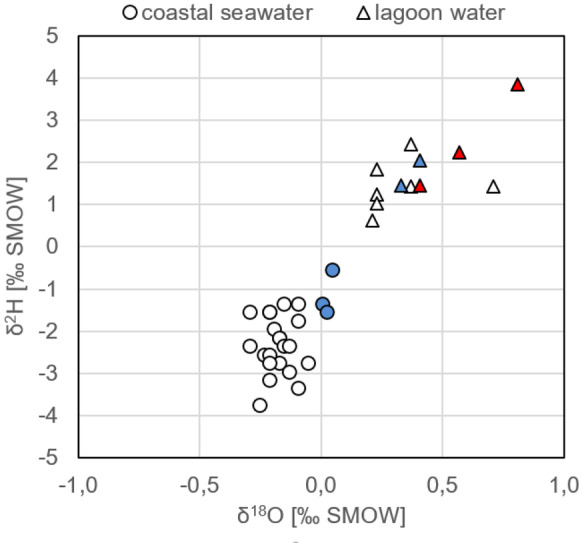
Surface water stable isotope signatures clustering in two groups, coastal sea and lagoon; the red triangles illustrate the water samples taken at the head of the lagoon, the blue triangles the samples taken close to its mouth; the blue circles indicate the seawater samples taken close to the lagoon’s mouth

**Fig. 7:**
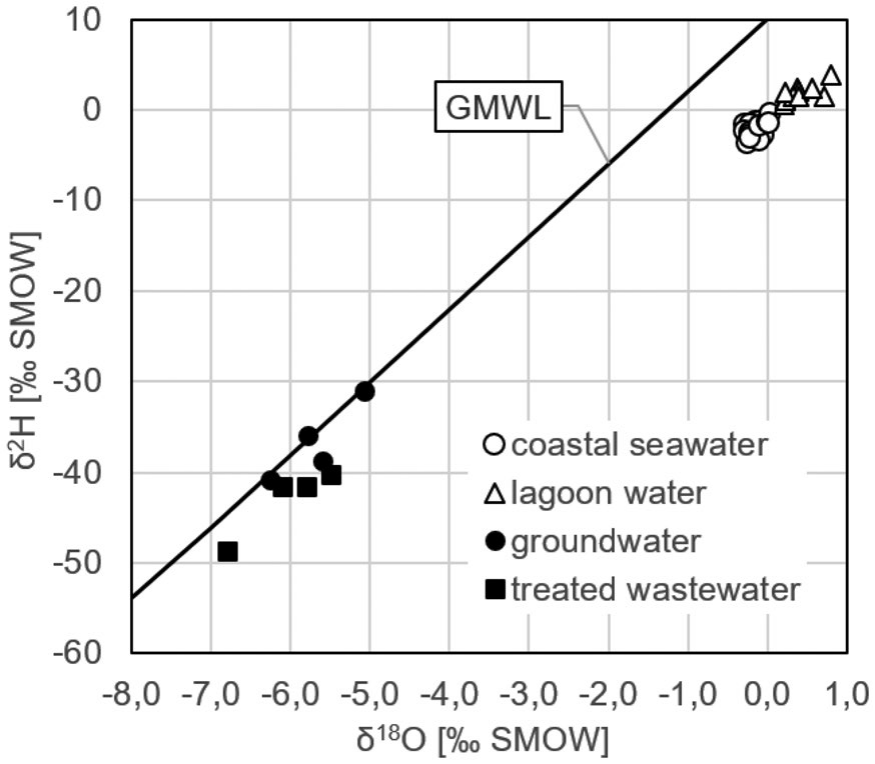
All water stable isotope signatures incl. groundwater and treated wastewater (data for the latter originating from Gilabert-Alarcón et al., 2018)

As a result of evaporation, the water stable isotope signatures of the collected seawater samples cluster within two distinct groups (Fig. 6): (1) The samples taken in the Bahia de Todos Santos coastal sea (displayed as circles) show a δ^2^H/δ^18^O range typical for the open ocean. The three seawater samples that were taken closest to the mouth of the lagoon are isotopically heaviest because of the discharging (evaporation impacted) lagoon water (blue circles in Fig. 6). (2) All water samples taken in the lagoon (displayed as triangles) are isotopically heavier than Standard Mean Ocean Water (SMOW; i.e. heavier than δ^2^H = 0‰/δ^18^O = 0‰). That reveals a strong evaporation impact on the lagoon water. The admixture of isotopically light discharged groundwater (incl. treated wastewater; Fig. 7; the displayed groundwater values originate from Gilabert-Alarcón et al., 2018, and another previously unpublished dataset) does not compensate the evaporation-caused isotopic shift towards heavier signatures within the lagoon. In fact, the three samples that were taken at the head of the lagoon (red triangles in Fig. 6) are isotopically heaviest because of the most intense evaporation impact (and the least impact of tidal flushing) there. The six samples that were taken in the centre of the lagoon (white triangles in Fig. 6) plot scattered. Generally, the samples that were taken closer to the lagoon’s mouth display lighter values than the ones that were taken closer to its head. However, besides the evaporation effect, the discharge of isotopically light SGD in subsections S3 and S4 has impact on the water stable isotope signatures of the samples taken there. This is also illustrated by the two samples taken at the lagoon’s mouth (blue triangles in Fig. 6), which plot in the middle of the lagoon cluster. Their position in the diagram indicates that they are, on the one hand, moderately impacted by evaporation but, on the other hand, not as strongly influenced by SGD as the samples taken in subsections S3 and S4.

## Conclusions

The combined evaluation of the processed tracer data allowed general conclusions regarding the suitability of the applied approach for SGD investigations in shallow coastal lagoons. On the one hand, the results confirm the general applicability of a radon mass balance for proving/disproving SGD occurrence. Whilst no groundwater discharge was traced in the coastal sea, SGD was proven within the lagoon. Here, areas of most intense SGD were localised and the related SGD flux rates quantified. On the other hand, the study revealed that water stable isotope and salinity data, which have been successfully applied in other SGD studies (e.g. Schubert et al, 2015), are of limited or no use at all in case of lagoons or estuaries that are highly impacted by evaporation. A further general result is that the tidal impact on lagoon water bodies can be levelled out by adapting the sampling schedule and cruise track to the tidal cycle, i.e. by mapping the profile of interest twice, once with the tide being rather low and once whilst it is rather high.

Furthermore, the study revealed site-specific insights. In the specific case of Estero de Punta Banda, the impact of evaporation is particularly intense and completely masks the signal of discharging isotopically light and fresh groundwater, thus limiting the applicability of salinity and stable isotope data as SGD indicators. Still, the joint evaluation of the radon, salinity and stable isotope datasets allowed locating the zone of influence of the tidal current within the lagoon: the difference in salinity between onward and backward cruise is most pronounced within the close-to-mouth subsections S1 and S2; the isotopically heaviest water was detected at the head of the lagoon indicating the least tidal flushing there. The detected radium ages complement the other tracer data reasonably. The lowest SGD residence times in the lagoon were found where (i) high salinity data indicate the least pronounced admixture of (“old”) seawater (at the head of the lagoon) and where (ii) high radon data indicate the most intense SGD (in subsection S3). Still, when the radium ages are evaluated in context with the other tracer data, it must be kept in mind that the radium activity ratios represent a 24-h sample period, i.e. cover (in contrast to all other data) two tidal cycles.

Summarising, it can be stated that whilst all individual sets of tracer data reveal reasonable information on their own, only a combined evaluation allows their comprehensive interpretation. Such combined evaluation permits compensating or allowing for tracer-specific limitations such as the impact of evaporation on salinity and stable isotopes as well as the tidal impact on radon.

## Data Availability

The datasets generated and/or analysed during the current study are available from the corresponding author on reasonable request.
